# Effect of Mixed Yeast Strains and Additives on the Quality of Long-Term Refrigerated Fermented Doughs

**DOI:** 10.3390/foods14050717

**Published:** 2025-02-20

**Authors:** Jun Liu, Lei Yang, Yingji Wang, Mengnan Wang, Qilong Qian, Lei Lou, Zhe Wu, Jiamin Zhu, Xiaoyu Fu, Jun Xing, Yixian Tu, Yun-Guo Liu

**Affiliations:** 1 Xinjiang Key Laboratory of Biological Resources and Genetics Engineering, College of Life Science and Technology, Xinjiang University, Urumqi 830046, China; 18099269493@163.com (L.Y.); wmn1529962@163.com (M.W.); 18290312429@163.com (L.L.); 18985229042@163.com (Z.W.); 15217433871@163.com (J.Z.); 18202380148@163.com (X.F.); xinjung@163.com (J.X.); daisy402@163.com (Y.T.); 2 Xinjiang Technical Institute of Physics and Chemistry, Chinese Academy of Sciences, Urumqi 830011, China; yjwang619@163.com; 3 College of Grain, Oil and Food Science and Material Reserve, Xinjiang Agricultural Vocational and Technical University, Urumqi 830001, China; qql0406@126.com; 4 College of Life Sciences, Linyi University, Linyi 276005, China

**Keywords:** mixed yeast strains, additives, long-term refrigeration, fermented doughs, quality

## Abstract

With a prolonged refrigeration time, dough becomes syruped and the gluten strength is weakened, which negatively affects the texture of the dough. At the same time, differences in the growth and metabolism of different yeasts lead to large differences in the physicochemical properties of the dough and the final quality of the product. Therefore, in this study, suitable additives and non-*Saccharomyces cerevisiae* were selected to minimize the effects of long-term refrigeration on the physicochemical properties and microstructure of fermented doughs. Compared to the control group without mixed yeast strains and additives, the fermentation properties, textural properties, dynamic rheology, starch crystallinity, protein structure, water distribution, and microstructure were investigated by mixed yeast strains and additives for 14 days of long-term refrigeration. The results showed that using mixed yeast strains (*Saccharomyces cerevisiae*: *Metschnikowia pulcherrima*; *Wickerhamomyces anomalous* = 0.46:0.27:0.27), α-amylase, diacetyl tartaric acid ester of mono(di)glycerides and polydextrose can avoid the excessive fermentation of refrigerated dough. In addition, mixed yeast strains and additives could maintain the orderliness of the secondary structure of gluten proteins, stabilize the microstructure of starch and gluten proteins, and reduce the migration and loss of water in the dough. This study clarified that mixed yeast strains and additives are conducive to prolonging the long-term refrigeration of dough, and could better maintain the quality of dough during long-term refrigeration. These results provide a theoretical basis for further research on the large-scale production of refrigerated fermented dough.

## 1. Introduction

Refrigerated dough is usually stored between 4 °C and 7 °C and then cooked into a finished product [1]. This technology avoids the need for high-power refrigeration equipment and inhibits microbial growth. It also inhibits the growth of microorganisms and extends the shelf life of the dough.

*Saccharomyces cerevisiae* is the primary fermenting strain used to ferment pasta products; this can make pasta products rise rapidly, but it is relatively homogeneous in terms of flavor [2,3]. Multi-strain fermentation can enrich pasta products’ flavor and nutrient content and change their waking speed. Current research on composite strains focuses on *Saccharomyces cerevisiae* mixed with lactic acid bacteria, *Saccharomyces cerevisiae* mixed with non-*Saccharomyces cerevisiae*, and lactic acid bacteria mixed with lactic acid. The mixed fermentation of Lactobacillus and Saccharomyces cerevisiae is more conducive to the formation of a larger specific volume, a lower hardness, and bread with better aroma characteristics [4,5]. A mixture of *Saccharomyces cerevisiae *and* Torulaspora delbrueckii* can be added to the dough to favor the growth of brewer’s yeast and the production of succinic acid, acetic acid, and essential amino acids in the fermented dough [6]. A mixture of multiple lactic acid bacteria produces more compounds such as aldehydes, esters, alcohols, and acids than a single lactic acid bacteria [7]. Some studies have addressed the compounding of *Saccharomyces cerevisiae* with non-*Saccharomyces cerevisiae*, finding that the addition of non-*Saccharomyces cerevisiae* is conducive to the prolongation of the storage period of the dough and the enrichment of the aroma and flavor components of fermented foods. Due to the differences in growth and metabolism of different yeasts, the physicochemical properties of the dough and the final quality of the product are quite different, so the selection of suitable non-*Saccharomyces cerevisiae* is the focus of the research on dough in the later stage of this study.

The current study shows that syruping and the weakening of the gluten strength affect the texture of dough under long-term refrigeration conditions. In this case, dough syruping is caused by xylanase enzymes degrading arabinoxylan and interfering with the cross-linking of arabinoxylan, thereby increasing the release of stagnant water from the dough [8]. The long-term refrigeration period can also cause the gluten strength of the dough to weaken over time. The problem of syruping refrigerated dough can be solved well by adding enzymes. For example, xylanase can effectively reduce the syruping of dough [9,10,11]. The addition of glucosyltransferase also enhances the cross-linking of the protein structure of the dough with arabinoxylan and reduces the syruping of the dough [11]. Additives such as emulsifiers (sucrose esters and sodium stearoyl lactylate), water-holding agents (guar gum and carboxymethyl cellulose), oxidizers (azodicarbonamide and calcium peroxide), and enzymes (xylanase and α-amylase) are synergistically combined in long-term refrigerated fermented doughs to solve the problem of the weakening of gluten proteins in long-term refrigerated fermented doughs and the syruping of the doughs [12]. The yeast fermentation process will be directed to convert phenolic acids to improve the quality of the dough [13]. However, during long-term refrigeration, the dough changes with its microstructure (e.g., the degradation of the gluten structure and aggregation of starch granules), which can accelerate the aging of the dough and affect the quality of the refrigerated dough [14].

In this study, in order to solve the above-mentioned problems, the commonly used method is to add improvers in order to optimize the properties of the stored dough, and the commonly used improvers are enzymes, moisture-retaining agents, and emulsifiers, etc. α-Amylase (AM) is one of the most widely used enzymes in the bakery industry. α-amylase can break down part of the starch to make the dough soft, increase the elongation, and obtain a large volume of bread with a fine structure. At the same time, AM can change the starch properties and make the aging effect of starch slow, prolonging the time that the bread remains soft, but the application should pay attention to controlling the amount of amylase; adding too much amylase much would make the volume of the bread small [15]. Polydextrose is a polymorph of glucose that is polymerized by high-temperature and low-pressure reactions in the presence of citric acid and sorbitol, a water-soluble dietary fiber [16]. Polydextrose has the functions of preventing the loss of water in dough and stabilizing dough properties. Diacetyl Tartaric Acid Esters of Monoglycerides (DATEM)) has strong emulsifying, dispersing, and aging-expanding effects, which can enhance the dough’s elasticity, toughness, and air-holding properties [17].

With the strong consumer demand for ready-to-eat bakery products and the implementation of front-of-the-store and back-of-the-plant processing methods in the bakery industry, long-term dough refrigeration (up to 14 days) is becoming increasingly urgent. The fermented dough can be refrigerated for an extended period, which can enhance the one-time production capacity of the processing plant, improve the distribution for stores at any time, and ensure the uniform quality and safety of each store’s products. This study will investigate the effects of mixed yeast strains and additives on refrigerated fermented dough by analyzing the changes in the physicochemical properties and microstructure of fermented dough during long-term refrigeration, which could provide a theoretical basis for applying long-term refrigerated fermented dough.

## 2. Materials and Methods

### 2.1. Materials

Yeast was donated by Xinjiang Desert Floating Fragrance Food Co., Ltd. (Urumqi, Xinjiang, China). *Wickerhamomyces anomalus* and *Metschnikowia pulcherrima* were purchased from Linyi Xiaolu Biotechnology Co., Ltd. (Linyi, Shandong, China). Yeast Extract Powder and Dextrose were purchased from Beijing Aoboshin Biotechnology Co., Ltd. (Biological Reagents, Beijing, China). Peptone was purchased from Beijing Solebao Science and Technology Co., Ltd. (Biological Reagents, Beijing, China). Marketed Tianshan Flour wheat flour was purchased from Xinjiang Tianshan Flour Co., Ltd. (Food-grade, Changji, Xinjiang, China). Edible salt was purchased from Xinjiang Salt Lake Salt Manufacturing Co., Ltd. (Food-grade, Urumqi, Xinjiang, China). Sugar was purchased from Xinjiang Green Plains Sugar Co., (Food-grade, Bayin’guoleng Mongol, Xinjiang, China). α-amylase was purchased from Shandong Loncote Enzyme Preparation Co., Ltd. (Food-grade, Linyi, Shandong, China). Polydextrose was purchased from Shandong Bailongchuangyuan Bio-technology Co., Ltd. (Food-grade, Jinan, Shandong, China). Diacetyl Tartaric Acid Esters of Monoglycerides were purchased from Henan Ornest Food Co., Ltd. (Food-grade, Zhengzhou, Henan, China). K_4[_Fe(CN)_6_] and KBr were purchased from Tianjin Huasheng Chemical Reagent Co., Ltd. (Analytical purity, Tianjin, China). ZnSO_4_ was purchased from Shanghai Shifeng Biotechnology Co., Ltd. (Analytical purity, Shanghai, China). NaOH granules were purchased from Tianjin Yongsheng Fine Chemical Co., Ltd. (Analytical purity, Tianjin, China). 3,5-dinitrosalicylic acid (DNS) was purchased from Beijing Kulaibo Technology Co., Ltd. (Beijing, China). K_2_Cr_2_O_7_ and H_2_SO_4_ were obtained from the Controlled Drugs Division, Laboratory, College of Life Sciences and Technology, Xinjiang University. The OCT frozen section embedding agent was purchased from SAKURA (Torance, CA, USA). Fluorescein isothiocyanate (FITC) and rhodamine B were purchased from Shanghai Yuanye Biotechnology Co., Ltd. (Analytical purity, Shanghai, China). Dimethyl sulfoxide solution (DMSO) was purchased from Tianjin Xinbute Chemical Co., Ltd. (Analytical purity, Tianjin, China).

### 2.2. Preparation of Refrigerated Fermented Dough

Yeast was added to YPD medium (Yeast Extract Peptone Dextrose Medium) and incubated at 28 °C and 180 r/min for 24 h; this was the first generation of activation. After the first generation of activation, the strain was added to YPD medium at 4% and incubated at 28 °C and 180 r/min for 24 h for the second strain activation. The number of strains from the second activation was diluted and adjusted to 10^7^ CFU/mL. Yeast was centrifuged at 4000 r/min for 5 min at 28 °C, and the slurry was washed twice in sterilized saline. Fresh yeast was obtained and added to the dough using a certain percentage of strain addition.

The experimental groupings are as follows. Control group (CG): The basic recipe of actual production dough was used, but with reduced yeast addition: 100 g of flour, 50 mL of water, 1.60 × 10^7^ CFU/g yeasts, 1.2 g of salt, 2.0 g of sugar, and 2.5 g of sunflower oil. Experimental group (EG): experimentally optimized recipe with 100 g of flour, 50 mL of water, and 1.60 × 10^7^ CFU/g yeasts (*Saccharomyces cerevisiae*: *Metschnikowia pulcherrima*; *Wickerhamomyces anomalous* = 0.46:0.27:0.27. The composite yeast strain addition ratios were determined by pre-experimentation and homogeneous design), 1.2 g of salt, 2.0 g of sugar, 2.5 g of sunflower oil, 20 mg of α-amylase, 2.4 g of polydextrose and 0.77 g of diacetyl tartaric acid ester of mono(di)glycerides (DATEM) (the critical food-grade additives were identified by a pre-experiment, and the range of their addition was determined by a one-way experiment and by a response surface experiment to determine the optimum amount of complex improver to be added in this experiment.). Flour was sifted into the mixer, and sugar and yeast were then mixed into the water and added to the flour; the mixer performed low-speed mixing for 5 min until the mixture was flocculent. Then, the salt and oil were mixed using medium-speed mixing for 15 min to make a dough. This was then taken out, kneaded, divided, weighed and packaged, before being put into the refrigerator freezer (4 °C) and stored for 0–14 days. Dough samples were taken from the control and modified groups on days 0, 7, and 14 for the corresponding experiments. Days 0, 7, and 14 of the control group are denoted as CG0, CG7, and CG14, respectively; days 0, 7, and 14 of the modified group are denoted as EG0, EG7, and EG14, respectively.

According to the experimental requirements, three pieces of dough weighing 350 g were removed from each group during the long-term refrigeration. Then 15 g of refrigerated small dough was taken and 135 mL of sterile saline was added to a micro masher (Shandong Dongfangke Instrument Co., Ltd., DS-1, Shandong, China) until homogenized. Separately, 30 g of dough was freeze-dried (Hangzhou Jutong Electronics Co., Ltd., FD-1A-50, Zhejiang, China), crushed in a mortar, and sieved through an 80-mesh sieve. The above-treated samples were set aside for subsequent experiments.

### 2.3. Fermentation Properties

#### 2.3.1. Leavening Ability and Gas-Holding Capacity

The leavening and gas-holding capacity are essential indicators of yeast quality and dough texture. First, 80 g of prepared dough was weighed into a 250 mL measuring cylinder and placed in the freezer at 4 °C. The height of the dough in the measuring cylinder (dough gas-holding capacity, v) and the increase in the height of the dough (yeast leavening ability, Δv) were recorded on days 0, 4, 7, 11, and 14, respectively.

#### 2.3.2. Reducing Sugar Content

**Figure 1:**
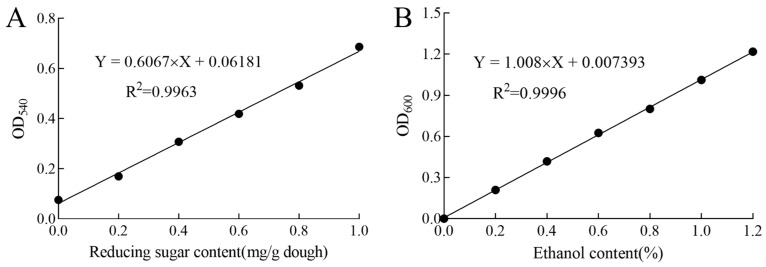
Standard curve of reducing sugar content (**A**) and ethanol content (**B**).

The Reduced sugar of the dough was measured according to the method of Saqib et al., with some modifications [18]. The preparation of the test solution: 10 g of dough was weighed into 90 mL of sterile saline, stirred in a magnetic stirrer for 30 min, and then centrifuged at 3500 r/min for 5 min. Then, 8 mL of the supernatant was taken after centrifugation, and 0.5 mL of 3.6% potassium ferrocyanide, 0.5 mL of 7.2% zinc sulfate, and 1 mL of 0.1 mol/L NaOH were added and mixed well, then centrifuged at 3500 r/min for 5 min to obtain the supernatant (test solution). A NaOH solution of 0.1 mol/L and 1 mL was mixed well and then centrifuged at 3500 r/min for 5 min to obtain the supernatant (liquid to be measured). The solution to be measured was taken and analyzed using the 3,5-dinitro salicylic acid spectrophotometric method. As shown in Figure 1A, glucose was used as a standard curve to determine the reducing sugar content of the refrigerated dough using an enzyme-labeling instrument (Meigu Molecular Instruments Co., Ltd., iD5, Shanghai, China).

#### 2.3.3. Ethanol Content

The pre-treated dough was dilute to the corresponding multiples; then, 2.5 mL of the reaction solution, 5 mL of 2% potassium dichromate solution, and 2.5 mL of concentrated sulfuric acid were added into a 10 mL colorimeter tube. The tube was plugged to seal it, and the solution was reacted for 10 min, shaken well, and cooled down to room temperature. Then, ultrapure water was used as a blank control and the absorbance was measured at 600 nm. As shown in Figure 1B, the ethanol content in the sample was calculated according to the standard curve.

#### 2.3.4. pH and Total Titratable Acidity (TTA)

The pH and TTA of the dough were measured according to the method of Xu et al. [19] The pH and TTA of the long-term refrigerated fermented dough were determined on days 0, 4, 7, 11, and 14.

### 2.4. Physical Properties of Dough

#### 2.4.1. Textural Properties of Dough

The texture of the sample was assessed using a Plus Texture Analyzer (Shanghai Baosheng Industrial Development Co., Ltd., TA.XTC-18, Shanghai, China) equipped with a cylindrical probe P/36, following a method outlined by Zhang et al., with some modifications [20]. The cylinder sample with a diameter of 40 mm and a height of 20 mm was cored out from the center part of the dough using a cork borer. A texture profile analysis was applied to the samples with a compression rate of 50%, a pre-test speed of 2.0 mm/s, a test speed of 2.0 mm/s, a post-test speed of 1.0 mm/s, and a trigger force of 5.0 g. The time interval between the two compressions was 5 s. The dough’s hardness, cohesion, elasticity, resilience, and adhesion were calculated according to the time–force deformation curve of the texture profile analysis. Each sample was measured in triplicate, and the measurements were averaged to present the texture quality of the sample.

#### 2.4.2. Dynamic Rheology Test

The rheological properties of the dough were examined using a dynamic shear rheometer (Thermo Fisher Scientific, Haake Mars 60, Waltham, MA, USA) with a parallel plate geometry (35 mm diameter, 1 mm height) according to the method of Lu et al., with some modifications [21]. The samples stored under refrigerated conditions for different days were weighed into 5 g samples. The rheological properties of the dough were determined using a rotational rheometer, and the dough was subjected to oscillatory frequency scanning tests at 25 °C. The dynamic frequency (0.1–10 Hz) tests were carried out at a strain of 0.5%, and the storage modulus (G’), loss modulus (G”), and loss tangent (tan δ = G”/G’) were recorded.

### 2.5. Physicochemical Properties of Dough

#### 2.5.1. X-Ray Diffraction

X-ray diffraction (XRD) (Bruker Corporation, D8 advance, Bremen, German) was used to analyze the crystal structure of the refrigerated fermented dough according to the method of Sun et al., with some modifications [22]. The samples were scanned at 40 kV and 30 mA in the range of 5–80° (2θ, 2.5°/min) with Cu-Kα radiation. The relative crystallinity of the samples was calculated using JADE software 9.0 (Materials Date Inc., Livermore, CA, USA). The relative crystallinity is the ratio of the sum of each peak area to the total area.

#### 2.5.2. Fourier Transform Infrared Spectroscopy (FT-IR)

The Fourier transform infrared spectra of the samples were obtained based on the method of Zhao et al. [23]. An FTIR spectrometer (Bruker Corporation, VERTEX 70 RAM II, German) was used to collect the FTIR spectra of gluten at wavenumbers ranging from 4000 cm^−1^ to 500 cm^−1^. In total, 32 scans were performed for each analysis at a resolution of 4 cm^−1^.

The secondary structures of gluten were measured by PeakFit version 4.12 software (SPSS Inc., Irvine, CA, USA). The secondary structure of the amide I (1600–1700 cm^−1^) interval was classified into β-sheet(1600–1640 cm^−1^), random curling (1640–1650 cm^−1^), α-helix (1650–1660 cm^−1^), and β-turn (1660–1700 cm^−1^), based on the spectra of the amide I (1600–1700 cm^−1^) interval. After performing processing and analysis using Peak Fit software, baseline correction, Gaussian smoothing, and deconvolution were performed to obtain secondary structure distribution maps with residuals greater than 0.99; this was undertaken to designate protein secondary structure characteristic peaks and to calculate the relative percentage of each structure.

#### 2.5.3. Low Field Nuclear Magnetic Resonance

The water distribution of the dough was determine using a ^1^H NMR spectrometer (Suzhou Niumag Analytical Instrument Corp., MesoMR23-060H-I, Suzhou, Jiangsu, China), following the protocol described by Wang et al. [24]. The middle 5 g of the sample dough was used to determine the transverse relaxation (T_2_) using the CPMG (Carr–Purcell–Meiboom–Gill pulse sequence) pulse sequence. The experimental parameters were as follows: SW = 200 kHz, SF = 21 MHz, RFD = 0.002 ms, RG1 = 20.0 db, TW = 2000 ms, NS = 8, TE = 0.200 ms and NECH = 1500. The inversion parameters were as follows: minimum relaxation time, 0.01 ms; maximum relaxation time, 10,000 ms; iterations, 100,000 times.

### 2.6. Microstructures of Dough

#### 2.6.1. Scanning Electron Microscopy (SEM)

The microstructure of the dough was investigated using a model SEM (Hitachi Limited., SU8010, Tokyo, Japan) with an acceleration voltage of 15 kV, according to the method of Liu et al. with minor modifications [25]. The sample was freeze-dried in a vacuum for 24 h. Subsequently, the freeze-dried dough powder was affixed to the loading platform and coated with gold through ion sputtering. The sample images were viewed at magnifications of 3000×. Representative micrographs were selected for illustration.

#### 2.6.2. Confocal Laser Scanning Microscope (CLSM)

The morphology of the frozen dough was observed by using a model CLSM (Nikon, A1RHD25, Tokyo, Japan), according to a method with minor modifications [26]. After the different treatments, the dough was frozen at −18 °C overnight, cut into cubes of about 1 cm and embedded with an embedding agent, sliced into 20 μm thick slices using a freezer slicer, and placed on slides. The cryosectioned samples were stained with 0.25% (*w*/*v*) fluorescein isothiocyanate (FITC) dimethyl sulfoxide solution (DMSO) and 0.025% (*w*/*v*) rhodamine B fluorescent dye. After 1 min, the samples were destained with deionized water, covered with coverslips, and were viewed under a confocal laser microscope. The excitation/emission wavelengths of the FITC and rhodamine B were 488/518 nm and 568/625 nm, respectively.

### 2.7. Statistical Analysis

All the experiments were performed in triplicates. Means and standard deviations were calculated. One-way analysis of variance and Duncan’s multiple range test were performed using SPSS 20.0 for Windows (SPSS Inc., Chicago, IL, USA). Duncan’s multiple range tests, at a significance level of *p* < 0.05, were performed to analyze the significance.

## 3. Results and Discussion

### 3.1. Effect of Mixed Yeast Strains and Additives on the Fermentation Properties of Dough on Long-Term Refrigeration

Yeast utilizes the nutrients in the dough to reproduce and metabolize the products. In the absence of oxygen, yeast converts glucose into CO_2_ and ethanol, and produces energy through glycolysis; under aerobic conditions, yeast can rapidly undergo germination and multiply, metabolizing glucose through aerobic respiration (glycolysis→tricarboxylic acid cycle) to produce CO_2_ and H_2_O [27]. The leavening ability (Δv) of dough reflects the yeast’s ability to produce gas for fermentation. Reducing sugars, the ethanol content, pH, and TTA, on the other hand, reflect the sugar consumption capacity and metabolite production capacity of the yeast in the dough.

Δv is the difference between the later volume capacity of the dough in the measuring cylinder and the former volume capacity. The gradual increase in Δv in the refrigerated dough is caused by the yeast beginning to utilize the material in the dough to produce carbon dioxide. When Δv decreases or becomes negative, this indicates that the refrigerated dough has collapsed. When Δv is zero, the volume before and after refrigerating the dough does not change. The refrigerated dough Δv in the CG increased, then decreased, and then increased again from CG0 to CG14.

**Figure 2:**
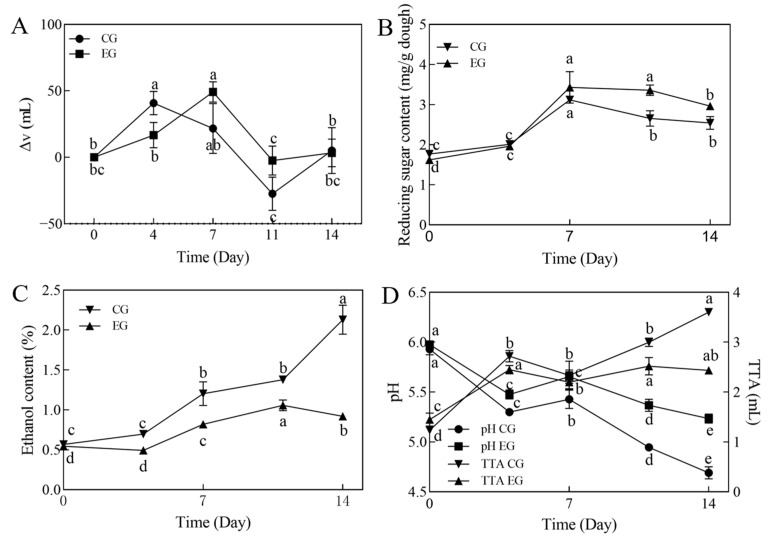
Changes in fermentability (**A**), reducing sugars (**B**), ethanol content (**C**), pH and TTA (**D**) of long-term refrigerated fermented doughs. Different letters (a–e) indicate statistically significant differences between groups at the same time point. The same letter indicates no significant difference and different letters indicate a significant difference. The error line for each data point indicates the standard deviation.

Moreover, the EG increases and decreases, finally converging to zero from EG0 to EG14. CG4 and EG7 are the Δv maxima for the CG and EG, respectively (Figure 2A). The results showed that the addition of a combination of non-*Saccharomyces Yeasts* with a low acid- and alcohol-producing capacity (*Metschnikowia pulcherrima* and *Wickerhamomyces anomalus*) and *S. cerevisiae* controlled the leavening power of the dough during long-term refrigeration, de-escalating the over-fermentation of the dough compared to *S. cerevisiae*.

Reducing sugar refers to the total content of all reducing sugars in the dough, including glucose, fructose, maltose, and others. From 0 to 14 days of long-term refrigeration, the reducing sugar content of the dough increased in both the CG and EG, but was consistently higher in the EG than in the CG (Figure 2B). This is due to the enzymatic degradation of starch by self-contained enzymes such as amylase and protease in the flour and by extracellular enzymes such as amylase and protease, which are produced by the added yeast during metabolism. The continuous production of acid by the yeast lowered the pH of the dough from 6.0 to 4.6, which affected the amylase and protease activities in the dough, increased the amylase activity in the dough, and promoted and inhibited the production of reducing sugars in the dough [28]. The low-temperature environment is also one of the important factors that causes a reduction in the sugar content, which not only affects the efficiency of yeast enzyme production and metabolite production but also attenuates enzyme activity. Therefore, the dough, under the combined influence of a low temperature and pH, inhibited the production of extracellular enzymes such as amylase and protease and acid metabolites, as well as the catabolic activity of the substrate, which in turn led to changes in the reducing sugar content of the dough. Long-term refrigeration is also accompanied by the yeast’s consumption of reducing sugars. The results showed that the EG was less capable of integrating the reducing sugar metabolism through the complexation of *S. cerevisiae* with *M. pulcherrima* and *W. anomalus* than the single *S. cerevisiae.* They also verified the changes in the leavening ability during cold storage in both groups.

During the long-term refrigeration process, the reproductive metabolism of the yeast caused a continuous increase in alcohol and acid production in both groups of dough, and the CG was higher than the EG; the pH of the CG and EG gradually decreased (Figure 2C,D). It has been shown that brewer’s yeast can catabolize glucose during metabolism to produce succinic and phenolic acids [13,29], ethanol, and carbon dioxide [30], which increase the organic acid content of the dough. In the composite yeast, *M. pulcherrima* is a low-fermentability yeast, so the yield of volatile acids would be lower under anaerobic conditions [31,32]. On the other hand, *W. anomalus* is characterized by acid tolerance, low water activity, high osmotic pressure, and anaerobicity, as well as the inhibition of fungi such as Aspergillus, Penicillium, and Fusarium, and bacteria such as Irwinia, Enterobacter, and Streptococcus [33,34]. Therefore, the accumulation of both ethanol and organic acids in the refrigerated dough of the EG was lower than that of the CG with a single yeast, and the dough of the improved group avoided the infestation of external heterotrophic bacteria and maintained a better state of organization at a relatively high pH value. From the changes in the fermentation characteristics of refrigerated dough at 0–14 days, it is clear that the changes in refrigerated dough at 0, 7, and 14 days are the main findings. Therefore, in a further in-depth study of the refrigerated dough at a later stage, 0, 7, and 14 days were taken as the primary collection and observation points.

### 3.2. Effect of Mixed Yeast Strains and Additives on Dough Properties on Long-Term Refrigeration

#### 3.2.1. Effect on the Gas-Holding Capacity of Long-Term Refrigerated Fermented Doughs

**Figure 3:**
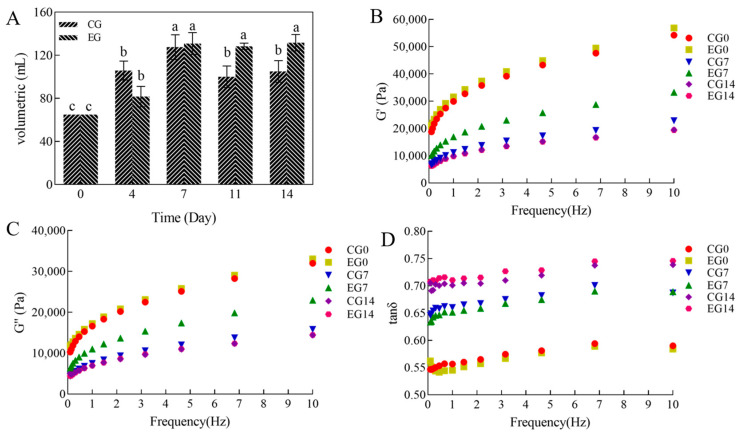
Changes in gas-holding capacity (**A**) and rheological properties (G’ (**B**), G” (**C**), tanδ (**D**)) of long-term refrigerated fermented doughs. Different letters (a–c) indicate statistically significant differences between groups at the same time point. The same letter indicates no significant difference and different letters indicate a significant difference. The error line at the top of each column in Figure 3A refers to the standard deviation.

During the long-term refrigeration period, the yeasts in the CG and EG continued to ferment. However, the gas-holding capacity of the dough varied, increasing and then decreasing in the CG and increasing and stabilizing in the EG. The gas-holding capacity of doughs is EG7max (130.80 mL) > CG7 max (127.50 mL) (Figure 3A). It was observed that the CG fermented rapidly from day 4 and had a much larger volume than the EG; by day 7, its swollen dough collapsed and had a smaller volume than the improved group on the same day. As yeast metabolism continued, the increase in ethanol content and decrease in pH in the exacerbated dough enhanced the protease activity, affecting the state of the gluten proteins and the structure of the proteins in the dough. In contrast, the EG slowed down the metabolism of the mixed yeast strains in the dough by effectively reducing the amount of *S. cerevisiae* added (only 46% of the CG) by compounding with *M. pulcherrima* and *W. anomalus*. Meanwhile, adding polydextrose in the EG better adsorbed the free water produced by metabolism in the dough. DATEM better facilitated the inclusion of fats and oils in the dough with other components. The addition of α-amylase, in turn, can supplement the deficiency in the fermentation capacity of the mixed yeast strains in the EG, maintaining a better gas production capacity.

#### 3.2.2. Effects on the Texture of Long-Term Refrigerated Fermented Doughs

The textural properties of dough, including its elasticity, cohesion, resilience, hardness, and adhesion, are essential indicators of its quality [35]. Positively related to dough quality are elasticity and resilience, and negatively related to dough quality are cohesion, hardness, and adhesion. As seen in Table 1, elasticity decreased in both the CG and EG as the cold storage duration increased, but it was significantly lower in the CG and higher in the EG than in the CG. The cohesion was increased in both groups at different refrigeration times, and there was no significant difference between the groups, but both EGs were lower than the corresponding CGs. Responsiveness increased and then decreased in the CG and did not change significantly in the EG. Hardness and adhesion (absolute values) were significantly lower in both the CG and EG, and both EGs were smaller than the CG. The three main reasons for the decrease in elasticity are recovery, hardness, and adhesion. Firstly, during long-term refrigeration, the continued fermentation of the yeast increases the CO_2_, ethanol, and organic acids, leading to a gradual buildup of starch in the dough. Secondly, the metabolites of the yeast destroy the hydrogen in the gluten proteins, resulting in a weakening of the gluten strength. Thirdly, it affects the adsorption of bound water with gluten proteins and starch in the dough [36]. Whereas, in the EG, DATEM stabilized the structure of gluten proteins [17], polydextrose enhanced the stability of water in the dough, and the addition of *M. pulcherrima* and *W. anomalus* slowed down the effect of yeast metabolites on the dough. Therefore, adding additives and mixed yeast strains prevented the deterioration of the textural properties of refrigerated dough and facilitated the storage of refrigerated dough.

#### 3.2.3. Effects on Rheological Properties in Long-Term Refrigerated Fermented Doughs

We analyzed the changes in the rheological properties of the refrigerated dough by determining the frequency scanning linear viscoelasticity. The rheological properties of wheat dough are usually characterized by three properties: the storage modulus G’ (elastic properties), loss modulus G” (viscous properties), and loss angle tan δ = G”/G’ (tan δ reflects the structural strength of the dough). A tan δ < 1 indicates that elastic properties predominate in the sample, and a tan δ > 1 indicates that viscous properties predominate in the sample [37,38]. The larger the tan δ, the weaker the gluten structure; the smaller the tan δ, the more robust the gluten structure [39].

The G’ and G” values of the dough increased with frequency within the range of 0.1–10 Hz, demonstrating a frequency-dependent behavior. Furthermore, the G’ value was higher than the G value, while tan δ consistently remained less than 1. These findings indicate that the doughs in each group exhibited properties more akin to elastic solids rather than viscous liquids. It was observed that the strength of the gluten structure gradually weakened as tan δ increased, and conversely, the gluten structure became stronger as tan δ decreased (Figure 3B–D). The refrigerated dough G’ and G” showed a decreasing trend, and tan δ showed an increasing trend in CG and EG from day 0 to day 14. This is consistent with changes in the gas-holding capacity and elasticity of the dough. The main reason for this change in trend is that yeast metabolite alcohols and acids go on to disrupt the binding capacity between starch and non-starch polysaccharides and depolymerize gluten proteins in long-term refrigerated fermented doughs [36]. The tan δ of EG0 and EG7 was smaller than that of CG0 and CG7. The results indicated that *M. pulcherrima* and *W. anomalus* slowed down yeast metabolism, α-amylase prevented the aging of starch in the dough [40], DATEM enhanced the interactions between starch, polysaccharides and proteins [17], and polydextrose prevented the conversion of bound water to free water [16]. Thus, including mixed yeast strains and compound additives resulted in a refrigerated dough with better elasticity and a more stable structure.

### 3.3. Effect of Mixed Yeast Strains and Additives on the Structural Properties of Dough on Long-Term Refrigeration

#### 3.3.1. Effect on Starch Crystallinity in Long-Term Refrigerated Fermented Doughs

**Figure 4:**
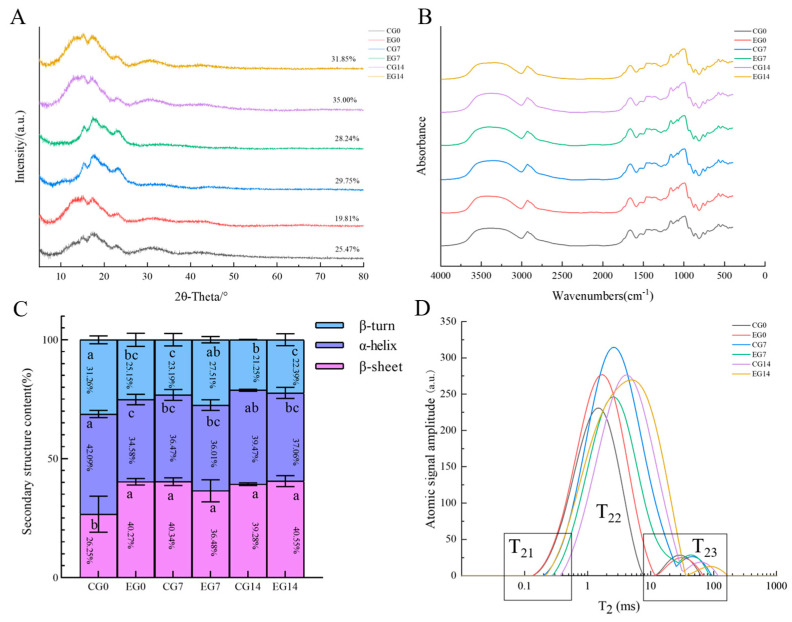
Changes in XRD (**A**), FT-IR (**B**), secondary structure content (**C**), and T_2_ relaxation time (**D**) of long-term refrigerated fermented doughs. The numbers on the curves in (**A**) indicate the crystallinity of the starch for which they are used. Different letters (a–c) indicate statistically significant differences between groups at the same time point. The same letter indicates no significant difference and different letters indicate a significant difference. The error line at the top of each column in Figure 4A refers to the standard deviation.

The starch in the dough undergoes aging regrowth after long-term refrigeration, and the relative crystallinity represents the degree of starch regrowth. The relative crystallinity of the starch was calculated using the ratio of the diffraction peak area to the total peak area. Specifically, the degree of starch regrowth is negatively correlated with the quality of the dough. It was observed that all the long-term refrigerated fermented doughs showed an A-type crystalline structure with characteristic peaks appearing at 15°, 17°, and 23° (2θ), consistent with the arrangement of wheat starch [41]. The relative crystallinity of the refrigerated dough increased from 21.14% to 56.08% during long-term refrigeration, with a gradual increase with time from CG0 to CG14. The refrigerated dough starch crystallinity ranged from 21.70% to 26.78% and was much lower than that of the CG from EG0 to EG14 (Figure 4A). The results showed that the aging of starch in the dough occurred in the CG under the action of yeast during long-term refrigeration, while the aging of starch was better controlled in the dough of the EG. This is mainly because the addition of α-amylase prevents the aging of starch in long-term refrigerated fermented doughs [40]. DATEM was closely related to the enhancement of the interaction between starch and gluten proteins and the prevention of the formation of crystalline regions of starch [17]. The mix of *M. pulcherrima* and *W. anomalus* with *S. cerevisiae* prevented the decomposition of organic acids’ starch molecules and increased the crystallization zone’s specific gravity. Therefore, adding mixed strains and additives better maintained the stability of the starch’s molecular structure, slowed down the aging of starch crystals, and helped maintain a better dough starch structure.

#### 3.3.2. Effects on Secondary Structure Changes of Proteins in Long-Term Refrigerated Fermented Doughs

Proteins’ secondary structure is crucial for forming the gluten protein matrix [42]. The α-helix structure is the main structure supporting peptides and contains a large number of hydrogen bonds [43,44]. The β-turn angle can determine the viscoelasticity of the dough [45]. β-sheet and irregularly coiled structures are associated with disorder in protein molecules.

The Fourier transform infrared spectroscopy (FTIR) plot in the 4000–500 cm^−1^ band shows that no new peaks were generated for each group of doughs, indicating that there was no new covalent bonding and that no new groups were generated in the long-term refrigerated fermented doughs during the storage process (Figure 4B) [46]. During long-term refrigeration, there were no random coil structures, suggesting that the disorder of proteins in brightly long-term refrigerated fermented doughs is mainly related to β-sheet (Figure 4C, *p* < 0.05). Between 0 and 14 days of long-term refrigeration, α-helix decreased from 42.09% to 39.28%, β-turn decreased from 31.26% to 21.25%, and β-sheet increased from 26.65% to 40.39% in the CG. The β-sheet, α-helix, and β-turn content of the EG were relatively stable (all changes were less than 1.6%), and there was a significant change in β-turn from EG7 to EG14. The results showed that the yeast in the dough led to a decrease in the plasticity and resistance of its gluten proteins to the ductility and a deterioration of its hydration under refrigerated conditions. Specifically, the yeast in the dough led to a decrease in the plasticity and resistance of its gluten proteins to the ductility, and a deterioration of its hydration under refrigerated conditions [11,47]. DATEM facilitated the relative stabilization of the dough’s secondary structure of gluten proteins. *M. pulcherrima* and *W. anomalus*, a complex of low-fermentability strains, controlled the metabolism of the yeast in the dough to avoid the destruction of the dough protein structure by excess metabolites. Therefore, mixed yeast strains and additives were beneficial in maintaining the order and hydrogen bonding of the secondary structure of gluten proteins in the dough.

#### 3.3.3. Effect on Distribution of Water in Long-Term Refrigerated Fermented Doughs

The distribution of water has an essential influence on the quality of refrigerated dough and the quality of the final product. The distribution of water and the binding state in food systems can be determined by using the spin–spin relaxation time (T_2_) and signal amplitude of protons [24,48]. According to the length of the relaxation time, it can be categorized into T_21_ (0.1–0.5 ms), T_22_ (8–40 ms), and T_23_ (70–300 ms), which indicate bound water, immobilized water, and free water, respectively, with T_22_ being the central peak [49].

The T_22_ peak has the highest signal, occupying about 90% of the peak area, indicating that the refrigerated dough system’s water is predominantly bound and does not easily flow. The T_22_ peak gradually moved closer to T_23_, indicating that the moisture in the dough gradually converted to free water from CG0 to CG14. Furthermore, from EG0 to EG14, the T_22_ peak moved to T_23_, where the T_21_ of EG14 had a more comprehensive range than that of EG7; the T_22_ peaks moved closer to T_21_ and T_23_, respectively, indicating that the moisture state of the dough was stable and that the strongly bound water was increased (Figure 4D). It was shown that *M. pulcherrima* and *W. anomalus* incorporation prevented the migration and loss of dough moisture due to yeast metabolites [50]. This was consistent with changes in the elasticity, adhesion, and rheological properties. This was because polydextrose binds water more tightly to starch and gluten proteins to reduce the free water in the dough. DATEM enhanced the starch–gluten protein interactions to avoid the conversion of bound water. Thus, including mixed strains and additives enhanced the water-binding capacity of the gluten structure, reduced the migration and loss of water from the dough, and improved the stability of the refrigerated dough.

### 3.4. Effect of Mixed Yeast Strains and Additives on the Microstructure of Long-Term Refrigerated Fermented Doughs

**Figure 5:**
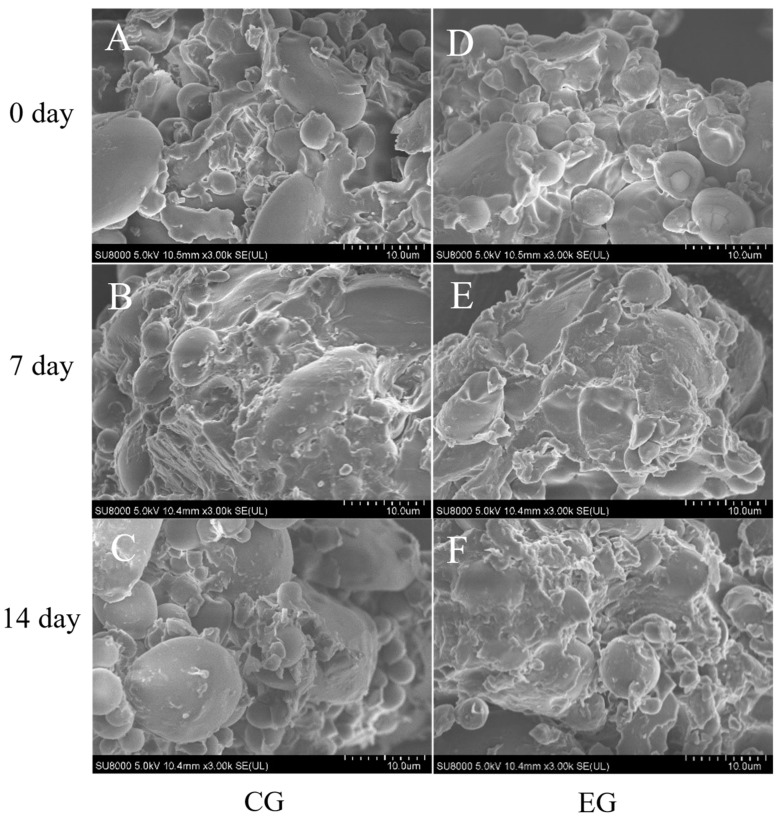
Changes in SEM (×3000) of long-term refrigerated fermented doughs. (**A**) CG0; (**B**) CG7; (**C**) CG14; (**D**) EG0; (**E**) EG7; (**F**) EG14.

**Figure 6:**
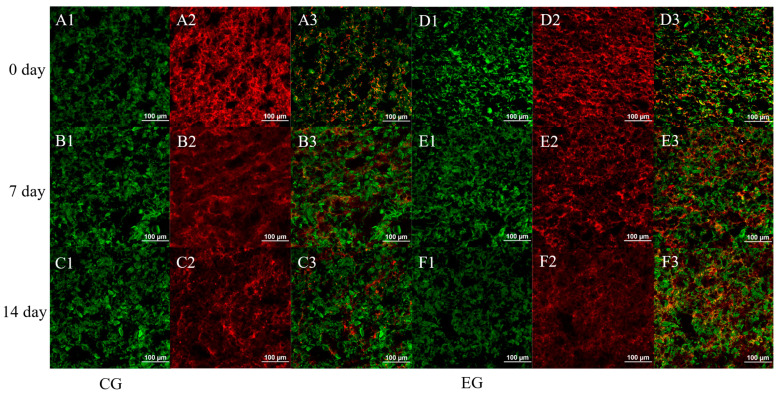
Changes in CLSM of long-term refrigerated fermented doughs. (**A**) CG0; (**B**) CG7; (**C**) CG14; (**D**) EG0, (**E**) EG7; (**F**) EG14. (**A1**,**B1**,**C1**,**D1**,**E1**,**F1**): the distribution of FITC-labeled starch in dough; (**A2**,**B2**,**C2**,**D2**,**E2**,**F2**): the distribution of Rhodamine B-labeled gluten; (**A3**,**B3**,**C3**,**D3**,**E3**,**F3**): FITC and Rhodamine B-labeled distribution of starch and gluten proteins together.

The SEM microstructural changes in the refrigerated dough are shown in Figure 5. The dough is a complex mixture in which the gluten forms a continuous three-dimensional network structure that encases the starch granules [51]. The voids in the microstructure are due to gaseous carbon dioxide produced by the yeast. CG0 and EG0 starch-sized granules were uniformly distributed in the gluten network structure (Figure 5A,D). This shows that all groups of doughs had a uniform distribution of starch and gluten structure on day 0 [52]. CG7 starch granules gradually aggregated and encapsulated within the gluten protein structure, and the CG14 gluten network structure loosened and receded, accompanied by exposed starch granules (Figure 5B,C). The EG7 and EG14 starch granules remained encapsulated with the gluten proteins and maintained a better state (Figure 5E,F). As seen in Figure 6, at specific excitation wavelengths, the gluten network structure and starch granules bound to rhodamine B and fluorescein isothiocyanate (FITC) dyes, respectively, each showing red and green colors, and the two macromolecules cross-linked to yellow [53]. As seen from CG0 to CG14, the red color in the field of view diminishes, and the yellow color becomes less, indicating a reduced dough protein structure and a weaker cross-link between the starch and proteins in the control group (Figure 6A–C). The dough gap increased in the experimental group, and the starch and protein structures were stably cross-linked and uniformly distributed (with yellow cross-link) from EG0 to EG14 (Figure 6D–F). This was because the metabolites of *S. cerevisiae* in the CG led to the hydrolysis of the gluten protein structure, and the aggregation of starch de-exacerbated the flaccidity of the gluten structure in the dough [14]. Incorporating *M. pulcherrima* and *W. anomalus* reduced the damage caused to starch and protein by yeast metabolites, and DATEM facilitated the interaction between starch and gluten network protein structures (with yellow cross-links). Therefore, mixed yeast strains and additives facilitated the interaction between starch and the gluten protein network structure, enhancing the protein gluten network structure and prolonging the long-term refrigeration period of the dough.

## 4. Conclusions

During short-term refrigeration, fermented doughs become limp and loose and cannot be stored longer. Therefore, in this paper, the textural properties and quality of the dough were improved by adding complex strains and improvers to extend the storage period of refrigerated fermented dough. This study found that the physical properties, structural characteristics, and microstructure of the dough in the control group (dough with only brewer’s yeast added) gradually deteriorated during the refrigerated storage period, and the phenomena of water analysis and structural limpness were obvious. The quality of the refrigerated dough in the experimental group was improved on day 14 due to the addition of the complex strain and the improver. Adding *M. pulcherrima* and *W. anomalus* effectively slowed down the fermentation rate of yeast in the dough during the refrigeration process, reduced the accumulation of undesirable metabolites, and prevented the deterioration of its physicochemical properties. This study also showed that additives such as α-amylase, diacetyl tartaric acid mono-diglycerides, and polydextrose helped to compensate for the lack of fermentation power in the non-brewer’s yeasts, which led to the stabilization of the distribution of the dough moisture and the gluten network structure during the cold storage process. These findings demonstrate that the quality of refrigerated dough can be maintained more effectively by using complex strains and improvers, which provides important theoretical support for the large-scale production of refrigerated fermented dough in the baking industry. Since fermented dough is mainly composed of various microorganisms, such as yeast and lactic acid bacteria, the compounding of various microorganisms, such as lactic acid bacteria, could be evaluated based on this study at a later stage. The mechanism of action of the supplemental enzymes and additives in fermented dough that improve its refrigerated quality can be further investigated by multi-omics techniques.

## Figures and Tables

**Table 1:** Changes in the textural properties of long-term refrigerated fermented doughs.

Groups	Textural Properties				
	Elasticity	Cohesion	Resilience	Hardness	Adhesion
CG0	0.94 ± 0.01 ^a^	0.77 ± 0.03	0.11 ± 0.00 ^c^	980.29 ± 12.23 ^a^	−1596.74 ± 6.63 ^e^
EG0	0.94 ± 0.00 ^a^	0.73 ± 0.03	0.13 ± 0.01 ^b^	916.37 ± 56.56 ^a^	−1011.96 ± 17.37 ^d^
CG7	0.93 ± 0.00 ^ab^	0.82 ± 0.04	0.14 ± 0.01 ^ab^	650.82 ± 29.20 ^b^	−594.55 ± 5.81 ^b^
EG7	0.94 ± 0.00 ^a^	0.78 ± 0.02	0.14 ± 0.01 ^ab^	546.89 ± 17.44 ^cd^	−467.70 ± 18.23 ^a^
CG14	0.90 ± 0.01 ^c^	0.79 ± 0.02	0.11 ± 0.00 ^c^	488.99 ± 30.90 ^de^	−659.95 ± 39.74 ^c^
EG14	0.92 ± 0.01 ^b^	0.78 ± 0.01	0.14 ± 0.00 ^ab^	411.92 ± 21.27 ^e^	−554.49 ± 29.80 ^b^

Note: Each value is expressed as mean ± standard deviation. Values in the same row with different superscript lowercase letters indicate significant differences (*p* < 0.05).

## Data Availability

The original contributions presented in the study are included in the article; further inquiries can be directed to the corresponding authors.
